# Enhancing hypertension detection and control through a hypertension certification program for pharmacists: A cluster randomized trial (The R_x_PATH Study)

**DOI:** 10.1177/17151635241254089

**Published:** 2024-05-31

**Authors:** Kaitlyn E. Watson, Jonathan C.H. Chan, Bo Pan, Yazid N. Al Hamarneh, Ross T. Tsuyuki

**Affiliations:** EPICORE Centre, Department of Medicine, University of Alberta, Edmonton, Alberta; EPICORE Centre, Department of Medicine, University of Alberta, Edmonton, Alberta; EPICORE Centre, Department of Medicine, University of Alberta, Edmonton, Alberta; EPICORE Centre, Department of Medicine, University of Alberta, Edmonton, Alberta; Department of Pharmacology, University of Alberta, Edmonton, Alberta; EPICORE Centre, Department of Medicine, University of Alberta, Edmonton, Alberta

## Abstract

**Importance:**

We designed an online educational program for primary care health care providers, the Hypertension Canada Professional Certification Program (HC-PCP), based upon its 2020 guidelines.

**Objective:**

The objective was to determine the effect of the HC-PCP, taken by pharmacists, on systolic blood pressure (BP) in patients with poorly controlled hypertension.

**Design:**

Stepped wedge cluster randomized trial (unit of randomization was the pharmacy).

**Participants:**

Patients with poorly controlled hypertension (BP >140/90 mmHg or >130/80 mmHg [diabetes]) in community pharmacies in Alberta, Canada, were recruited by their pharmacist.

**Intervention:**

Pharmacists completed the HC-PCP program, then provided care to their patients with poorly controlled hypertension according to what they learned in the course.

**Control:**

Pharmacists were given a copy of the Hypertension Canada guidelines and provided their usual care to their patients prior to undertaking the HC-PCP later.

**Main outcome and measure:**

The primary outcome was a difference in change in systolic BP at 3 months between groups, while the secondary outcome was patient satisfaction with using the Consultation Satisfaction Questionnaire.

**Results:**

We enrolled 890 patients from 59 pharmacies (including 104 pharmacists). Using a linear mixed-effect model with BP reduction as the dependent variable and independent variables of treatment allocation, baseline BP, site effect and patient effect, the intervention was associated with a 4.76 mmHg (95% confidence interval, 2.02–7.50, *p* < 0.0001) systolic BP reduction at 3 months. Patient satisfaction with using the Consultation Satisfaction Questionnaire was high at 75.9 (/90).

**Conclusion and relevance:**

Most educational programs are not evaluated at the patient care level. The HC-PCP taken by pharmacists resulted in a 4.76 mmHg systolic BP reduction in their patients over 3 months. This would have major implications for public health, reducing heart disease, stroke and kidney failure.

## Graphical abstract

**Graphical Abstract:**
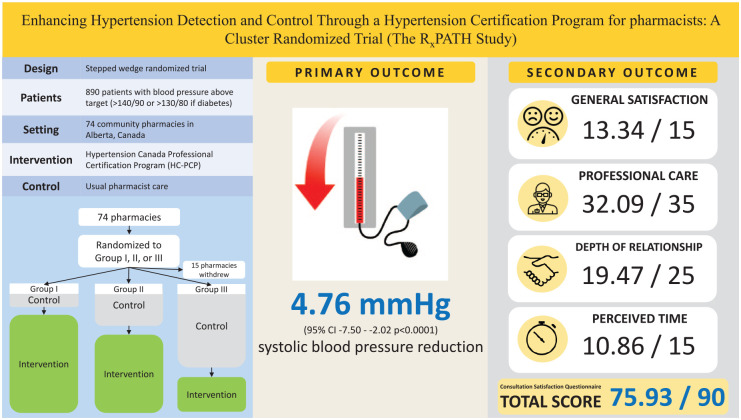

**Knowledge into Practice**

- Educational programs designed for health care professionals are often not evaluated at the patient care level to determine impact on patient outcomes.
- Pharmacists completed the Hypertension Canada Professional Certification Program (HC-PCP) and then provided care according to the program and guideline recommendations.
- The HC-PCP taken by pharmacists resulted in a 4.76 mmHg greater systolic blood pressure reduction in their patients over 3 months, compared to usual care.

## Introduction

Hypertension control remains a high priority to prevent complications, disability and death. In the mid-2000s, Canada emerged as a leader in hypertension care, with high national treatment and control rates.^1^ Yet a recent national study identified that rates of awareness, treatment and control are deteriorating.^1^ Hypertension detection and management is the core focus of many organizations, including Hypertension Canada (HC). HC provides up-to-date, evidence-based guidelines.^2^ However, the uptake of these guidelines and implementation into primary care providers’ clinical practice remains challenging.^3^

This challenge is not unique to hypertension, with research suggesting the uptake and incorporation of clinical practice guidelines into practice is limited for many chronic conditions.^3-6^ Indeed, it has been reported that 30% to 40% of patients receive treatment that is not based on evidence and guidelines.^4^ Barriers to implementation of hypertension treatment guidelines into practice are related to the knowledge, behaviour and attitudes of health care providers.^3^

**Mise En Pratique Des Connaissances**

- Les programmes éducatifs conçus pour les professionnels de la santé ne sont souvent pas évalués sur le plan des soins aux patients afin de déterminer l’impact sur les résultats pour les patients.
- Les pharmaciens ont suivi le Programme de certification professionnelle d’Hypertension Canada (PCP) et ont ensuite prodigué des soins conformément aux recommandations du programme et de ses lignes directrices.
- Le PCP suivi par les pharmaciens a permis une réduction de la PA systolique de 4,76 mmHg de plus chez leurs patients sur une période de 3 mois, par rapport aux soins habituels.

To help address these challenges in hypertension management, we created a novel evidence-based program, the Hypertension Canada Professional Certification Program (HC-PCP), which aims to improve the integration of the current Canadian hypertension guidelines into practice for primary health care providers (e.g., pharmacists, physicians, nurses) through education and training.^7^ The HC-PCP was created and constructed around core competencies that were developed by hypertension experts and agreed upon by primary care providers.^7^ The HC-PCP incorporates self-directed learning with immediate and delayed feedback from hypertension experts.^7^ There are 4 online learning modules with a quiz to assess knowledge, an evaluation of correct blood pressure (BP) measurement technique through submission of a video and expert evaluation of hypertension management by participants submitting 3 of their patient cases for feedback. Once health care providers successfully pass all the components, they are provided with their certification in hypertension management. The HC-PCP has been designed for primary health care professionals, with pharmacists chosen as the initial group to undertake the program.

Patients with chronic conditions like diabetes and hypertension see their community pharmacist more frequently than any other health care professional.^8^ Such frequent encounters may be the key to better identification and management of these conditions, as ease and timeliness of access were highlighted by patients as major attributes for using clinical pharmacy services.^9^ In Alberta, pharmacists have the broadest scope of practice in the world, which includes independent prescribing and ordering and interpreting laboratory tests.^10^ The ease of access combined with the broad scope of practice puts pharmacists in a prime position to systematically identify patients with hypertension and contribute to their treatment and management. Recent multicentre randomized trials have demonstrated that pharmacist interventions do not only improve patients’ clinical outcomes (BP and cardiovascular risk) but also reduce health care costs.^11-14^ Indeed, it has been reported that pharmacists’ care was associated with an 18.3 mmHg reduction in systolic BP,^12^ 21% reduction in cardiovascular risk^11^ and an estimated $15.7 billion in cost savings in Canada over a 30-year time horizon (using a conservative model for pharmacist care in hypertension).^14^

While there is strong evidence for the impact of pharmacist care in hypertension, the uptake and implementation of this evidence is lacking. Therefore, we conducted this study to investigate the effect of the HC-PCP taken by pharmacists on systolic BP reduction in patients with hypertension whose blood pressure is not within target, compared to usual care.

## Material and methods

### Study design

**Figure 1:**
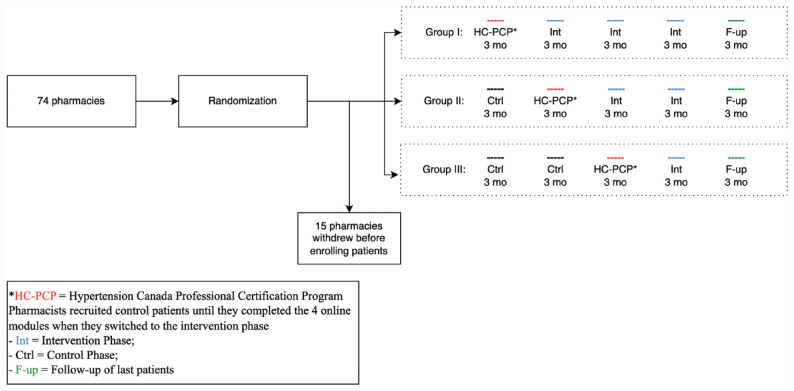
Stepped wedge cluster randomized design

We employed a stepped wedge cluster randomized trial with the pharmacy as the unit of randomization (Figure 1). Such designs are commonly used for health services intervention studies, as they allow for the high degree of causal inference of a randomized trial, yet allow all pharmacists to receive the intervention training.

### Recruitment and data collection

This study was conducted in community pharmacies across the province of Alberta, Canada. Participating pharmacists were identified through the Alberta Pharmacists Association and social media. Each pharmacy was asked to have at least 2 pharmacists who would participate in the study.

Patients were recruited on a continuous basis by the participating Alberta community pharmacists. The patient eligibility criteria included adults (18 years or older) with BP that is not within target (>140/90 mmHg or >130/80 mmHg if they have diabetes). For those who have been previously diagnosed with hypertension, the average of 3 readings was required to be considered not within target. For patients without a previous hypertension diagnosis, the average of 3 readings not within target on 2 consecutive visits (based on the HC guidelines for hypertension diagnosis) was required.^2^

Patients were excluded from the study if they

• Were unwilling to participate/sign a consent form
• Were unwilling or unable to participate in regular follow-up visits
• Had a current hypertensive urgency or emergency (systolic BP >180 mmHg or diastolic BP >120 mmHg with or without symptoms)
• Were known to have secondary causes of their hypertension (e.g., pregnancy)

All BP measurements were conducted using correct Automated Office Blood Pressure (AOBP) measurement on the study-provided WatchBP Office AFIB device (Microlife, Widnau, Switzerland) or provided home BP monitoring readings.

The unit of randomization was clustered at the pharmacy level. Pharmacies were randomized (stratified by urban vs rural location) to 1 of 3 groups. Group I started in the control phase enrolling control patients while they were completing the HC-PCP. Once they completed the 4 online modules, they switched to the intervention phase. Groups II and III served as controls (for varying durations) before completing the HC-PCP and switching to the intervention (Figure 1).

The pharmacists were blinded to the stepped wedge design and were informed the delayed start for groups II and III was due to capacity in the program. The statistician was blinded in the analysis as to the groupings. Pharmacists in the intervention groups could not be blinded. However, pharmacists were not informed about the cluster randomization design of the study. They were told that they would be contacted about *when* they would be enrolled into the HC-PCP training. During the control period, pharmacists were told that their patients would be enrolled in an observational hypertension registry to obtain baseline readings and to prepare for the intervention phase.

### Intervention and control

During the control period, pharmacists received an introductory workshop based upon the 2020 HC guidelines,^2^ focusing on screening for hypertension. All patients with BP not within target were entered into the study database and served as the control group. No specific interventions or follow-up were mandated other than usual pharmacist care. The intervention period started with pharmacists being invited to complete the HC-PCP within a 3-month time frame, and then they provided care based on the program to their patients. The program intervention included performing a cardiovascular risk assessment, prescribing or adjusting antihypertensive medications and conducting monthly follow-up visits according to the HC guidelines. Pharmacists continued recruiting control patients until they completed the 4 online modules. Once they completed the modules, pharmacists switched to the intervention phase and submitted their case management for expert evaluation to pass the program.

All patients were followed up for a minimum of 3 months but continued until the end of the trial (total of 15 months). Patients in the intervention phase were provided with follow-up visits according to the guidelines (e.g., monthly for medication/dose changes). Follow-up frequency was not specified in the control group. Both intervention and control patients continued to see their family physician as per usual practices.

### Outcomes and interpretation

Primary outcome was the difference in change (from baseline to 3-month follow-up) in systolic BP between intervention and control patients. Secondary outcome was patient satisfaction using the Consultation Satisfaction Questionnaire (CSQ), which is a validated questionnaire that has been widely used to rate the care from health care providers (e.g., general practitioners and nurse practitioners).^15,16^ The questionnaire consists of a general satisfaction section and 3 subsections: (1) the professional aspects of the consultation, (2) the depth of the patient’s relationship with the pharmacist and (3) the perceived length of the consultation.^17^ The CSQ was voluntary and completed by patients at the conclusion of the study period. All patients who completed the CSQ had received the intervention due to the stepped wedge design. Thus, there is no comparison measure for the CSQ.

### Context

Patient recruitment was supposed to take place in March 2020. Due to the global COVID-19 pandemic, the start of the study was postponed. However, because of the funding conditions, the study had to be started in 2020. Thus, the study began in September 2020 when the COVID-19 vaccine became readily available in Alberta, and in-person physical assessments were allowed. Resources and materials were provided to pharmacists to take appropriate measures, including disinfecting wipes to clean the BP device between patients and links to additional guidance on personal protective equipment and risk assessment.

Because of the ongoing nature of the pandemic, some study parameters had to be adapted. First, many patients were still uncomfortable coming into pharmacies to have their BP measured, so it was determined that pharmacists could complete study visits via telehealth services and record a home BP measurement (HBPM) after teaching the patient the correct HBPM technique according to the HC guidelines. Second, some pharmacists struggled to complete the HC-PCP within the specified 3-month period with their additional COVID-19–related roles and responsibilities (e.g., screening, vaccinations, testing, etc.). To address this, additional time was allowed for pharmacists to complete the HC-PCP. For analysis, it was determined that the date at which they switched from the control to intervention period was the date they finished the fourth module quiz of the HC-PCP and began the case evaluation of the HC-PCP in which they were required to demonstrate examples of providing the intervention for feedback from hypertension experts to pass and complete the program. This led to some overlap between groups finishing the HC-PCP and groups starting it. Because of these unavoidable adaptations to the study protocol, the original data analysis plan using the stepped wedge design could not be completed. Instead, we compared the overall intervention data to the overall control data regardless of the time of delivery.

Originally, 150 pharmacists from 74 pharmacies were enrolled in the study. Fifteen pharmacies withdrew at the beginning of the study before patient recruitment due to their COVID-19 pandemic responsibilities (Figure 1). Fifty-nine of the 104 pharmacists who continued in the study completed the HC-PCP and received their certificates in hypertension management. The other 45 dropped out of the study and did not complete the HC-PCP (which was the intervention). As such, their patients remained in the control group.

### Sample size and data analysis

The sample size estimation in the proposed cluster randomized trial was performed by R Package “CRTSize.”^18^ To detect a minimum difference of 7 mmHg in the change of systolic BP between the control and treatment groups, a sample size of 40 clusters with 20 patients per cluster (800 in total) is needed to ensure 80% power, given an alpha of 0.05, an estimated standard deviation of 20 and an interclass correlation coefficient of 0.1. To account for dropouts and losses to follow-up and stratification, we aimed to recruit 78 pharmacies (26 per group).

Statistical analysis was performed using R 3.4.0 (Vienna, Austria; https://www.R-project.org/) and SAS 9.4 software (SAS Institute, Cary, NC, USA). Patients’ demographic information and clinical characteristics were examined with descriptive statistics, using frequency (percentage) for categorical variables and mean (standard derivation), median (interquartile range) and range for continuous variables as appropriate. Univariate level analysis was conducted using hypothesis testing to explore whether there are any statistical differences between the groups. Chi-square or Fisher tests (when small frequencies are present) were used for categorical variables and *t-*test or Wilcoxon rank-sum tests (when data are heavily skewed) for continuous variables (assumptions of statistical tests were checked ahead of conducting the test). The primary outcome, difference in systolic BP reduction between the intervention and control group, was analyzed using analysis of covariance (ANCOVA) while adjusting for the baseline BP. Regression analysis was performed to quantify the impact of intervention. The potential impact of intervention was examined by linear mixed-effect regression models while adjusting for the site and patient individual effect. The secondary outcome, CSQ, was analyzed by descriptive statistics, presenting the frequency (%) for each question.

### Ethics approval and trial registration

This trial was approved by the University of Alberta health research ethics board (Pro00090012), ClinicalTrials.gov Identifier: NCT03965104.

## Results

**Figure 2:**
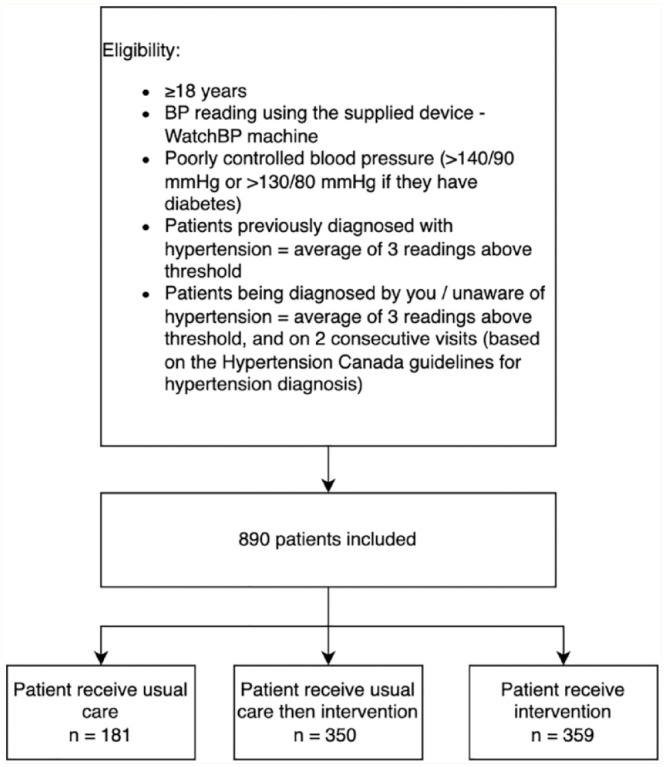
Study eligibility and patient enrollment

A total of 890 patients were recruited by 104 pharmacists at 59 pharmacies between September 2020 and February 2022 (Figure 2). Due to the continuous enrollment of patients in the stepped wedge design and with the COVID-19 pandemic context challenges described above, some of patients who were recruited in the control/training phase remained in the control group if the pharmacists did not complete HC-PCP training.

### Patient demographics

There was an almost even split between male and female patients (53.6% were male) (Table 1). The mean (SD) age was 61.6 (14.2) years, and most participants were white (78.4%). Almost half (48.3%) of the participants had dyslipidemia, 34.5% had diabetes and 10.3% had a diagnosis of chronic kidney disease. Most patients (81.6%) had an existing diagnosis of hypertension, while 18.4% received a new diagnosis during the study period (Table 1).

**Table 1:** Patient demographics

Characteristic	Control (*n* = 181)	Intervention (*n* = 359)	Control—Intervention (*n* = 350)	Total (*N* = 890)
Sex				
Male	94 (51.9)	187 (52.1)	196 (56.0)	477 (53.6)
Female	87 (48.1)	172 (47.9)	154 (44.0)	413 (46.4)
Age, mean (SD), y	61.8 (14.1)	60.7 (14.2)	62.4 (14.1)	61.6 (14.2)
Body mass index, mean (SD)	31.2 (7.2)	31.9 (7.6)	31.1 (7.0)	31.4 (7.3)
Ethnicity				
First Nations	8 (4.4)	13 (3.6)	19 (5.4)	40 (4.5)
Arab	4 (2.2)	4 (1.1)	3 (0.9)	11 (1.2)
Black	8 (4.4)	8 (2.2)	17 (4.9)	33 (3.7)
Caucasian	127 (70.2)	294 (81.9)	277 (79.1)	698 (78.4)
Hispanic	5 (2.8)	13 (3.6)	6 (1.7)	24 (2.7)
South Asian (Indian, Pakistani, Sri Lankan, Bangladeshi)	10 (5.5)	7 (2.0)	8 (2.3)	25 (2.8)
East Asian (Chinese, Korean, Japanese)	21 (11.6)	21 (5.9)	23 (6.6)	65 (7.3)
Other	1 (0.6)	2 (0.6)	3 (0.9)	6 (0.7)
Tobacco				
Current	31 (17.3) (*n* = 179)	56 (15.6) (*n* = 359)	63 (18.1) (*n* = 349)	150 (16.9) (*n* = 887)
Former	38 (21.2)	112 (31.2)	84 (24.2)	234 (26.4)
Never	110 (61.5)	191 (53.2)	202 (57.9)	503 (56.7)
Exercise				
Very active	15 (8.4)	41 (11.4)	31 (8.9)	87 (9.8)
Moderately active	62 (34.6)	121 (33.7)	112 (32.1)	295 (33.3)
No exercise	102 (57.0)	197 (54.9)	206 (59.0)	505 (56.9)
Diabetes	64 (35.4)	120 (33.4)	123 (35.1)	307 (34.5)
Chronic kidney disease	18 (9.9)	28 (7.8)	46 (13.1)	92 (10.3)
Dyslipidemia	93 (51.4)	178 (49.6)	159 (45.4)	430 (48.3)
Angina	12 (6.6)	7 (2.0)	13 (3.7)	32 (3.6)
Myocardial infarction	16 (8.8)	13 (3.6)	23 (6.6)	52 (5.8)
Hypertension diagnosis				
New	29 (16.0)	77 (21.5)	58 (16.6)	164 (18.4)
Already managed	152 (84.0)	282 (78.6)	292 (83.4)	726 (81.6)

Values are presented as number (%) unless otherwise indicated.

### Primary outcome

The primary outcome was the difference in change (from baseline to 3-month follow-up) in systolic BP between intervention and control patients. Using a linear mixed-effect model with BP as the dependent variable and independent variables of treatment allocation, baseline BP, site effect and patient effect, the intervention was associated with a 4.76 mmHg (95% confidence interval, 2.02–7.50, *p* < 0.0001) systolic BP reduction at 3 months.

### Secondary outcome

Patient satisfaction was evaluated using the CSQ. Patients were asked to complete the CSQ after their final visit, having received the intervention. There were 147 patients who completed the CSQ (Table 2). Patient satisfaction was high at a mean (SD) of 75.9 (7.7) out of 90 (a higher score indicates a higher level of satisfaction). Breaking down the total score, patients rated general satisfaction as 13.34/15, professional care as 32.09/35, depth of relationship as 19.47/25 and perceived time as 10.86/15.

**Table 2:** Patient satisfaction

Characteristic	Treatment, Mean (SD)
**Total score (out of 90)**	75.9 (7.7) (*n* = 147)
**General satisfaction**	
I am totally satisfied with my visit to this pharmacist.	4.8 (0.5) (*n* = 159)
Some things about my consultation with this pharmacist could have been better.	4.1 (0.9) (*n* = 158)
I am not completely satisfied with my visit to the pharmacist.	4.4 (0.9) (*n* = 158)
** Subtotal (out of 15)**	**13.3 (1.6) (*n* = 157)**
**Factor 1: Professional care**	
This pharmacist was very careful to check everything when examining me.	4.7 (0.5) (*n* = 157)
This pharmacist listened very carefully to what I had to say.	4.7 (0.5) (*n* = 159)
This pharmacist told me everything about my condition/medications.	4.6 (0.5) (*n* = 157)
I thought this pharmacist took note of me as a person.	4.6 (0.6) (*n* = 159)
I will follow this pharmacist’s advice because I think he/she is absolutely right.	4.6 (0.6) (*n* = 158)
This pharmacist was interested in me as a person, and not just my illness.	4.5 (0.6) (*n* = 159)
I understand my illness much better after seeing this pharmacist.	4.4 (0.7) (*n* = 159)
** Subtotal (out of 35)**	32.1 (2.8) (*n* = 155)
**Factor 2: Depth of relationship**	
There are somethings this pharmacist does not know about me.	3.5 (1.2) (*n* = 159)
This pharmacist knows all about me.	3.8 (1.0) (*n* = 159)
I felt that this pharmacist knew what I was thinking.	3.7 (0.8) (*n* = 158)
I felt I was able to tell this pharmacist everything.	4.6 (0.5) (*n* = 159)
I would find it difficult to tell this pharmacist about certain private issues.	3.9 (1.0) (*n* = 159)
** Subtotal (out of 25)**	19.5 (3.2) (*n* = 158)
**Factor 3: Perceived time**	
The time I was allowed to spend with the pharmacist was not long enough to deal with everything I wanted.	4.0 (1.0) (*n* = 158)
I wish it had been possible to spend a little longer with the pharmacist.	3.2 (1.1) (*n* = 154)
The time I was able to spend with this pharmacist was a bit too short.	3.7 (1.1) (*n* = 159)
** Subtotal (out of 15)**	10.9 (2.6) (*n* = 153)

## Discussion

Professional certification of pharmacists in hypertension management was associated with a significant reduction in their patients’ systolic BP when compared to usual care over 3 months. The intervention was also associated with high levels of patient satisfaction. To our knowledge, this is the first randomized trial demonstrating the impact of an educational program on patient-level outcomes in hypertension. Our study showed a 4.76 mmHg greater reduction in systolic BP compared to usual care—this highlights that a professional certification program undertaken by a health care provider can have a positive impact on patients. The HC-PCP is scalable to further improve detection and management of hypertension across Canada and could be implemented by all primary care health care providers, which would likely lead to better detection and control rates of BP across Canada.

Health care provider certification programs are not new to chronic conditions. For example, the Heart Failure Society of America has an examination process to certify health care providers (physicians, pharmacists, nurses) in recognition of their advanced expertise in heart failure.^19^ The American Heart Association provides certification at the health system level for hospital and outpatient clinic settings.^20^ In Canada, health care provider certification programs exist for other chronic conditions—in areas such as asthma, tobacco cessation, chronic obstructive pulmonary disease and diabetes.^21,22^ However, none of these programs have been evaluated at the patient level. Health care provider education programs are often evaluated at the individual pre/post knowledge level. For example, a 2018 feasibility study of a hypertension training program designed for nonphysicians found that a 5-day training program improved health care providers’ knowledge.^23^ Additionally, patient education programs were shown to improve patient knowledge, medication adherence and BP control.^24^ In other cardiovascular diseases, such as heart failure, different styles of patient educational programs have been evaluated. Empowerment-based self-care programs were found to be more effective than didactic programs to improve patients’ symptom perception and disease self-management.^25^

The HC-PCP is the first hypertension certification program available in Canada. Implementation of such programs in accordance with guideline-directed management of hypertension can improve hypertension control, detection, management and treatment. Further investigations of the HC-PCP and its impact on patients’ systolic BP may include the evaluation of the clinical effectiveness in other health care professionals (such as family physicians and nurse practitioners). Further evaluation of HC-PCP outside of the pandemic context with a longer follow-up duration may provide additional insights on the program’s impact within primary care from the community pharmacy perspective. Additionally, this study could open the door for evaluation studies of other health care provider–based certification programs on patient-oriented outcomes.

### Strengths and limitations

The R_x_PATH trial started in September 2020 during the height of the COVID-19 pandemic. Community pharmacies faced unprecedented staffing and workload challenges. With heavy workloads, it was difficult for pharmacists to complete the HC-PCP within the dedicated time period, which resulted in the withdrawal of 15 pharmacies before starting patient recruitment. Additionally, the COVID-19 pandemic presented challenges for this study, as patients were reluctant to visit pharmacies in person to have their BP measured. This resulted in adaptations to the study protocol allowing for home BP to be used (in place of an AOBP on the supplied WatchBP device) and telehealth appointments for patient follow-up visits. This resulted in the limitation that home BP is not the same (typically lower) as office BP.^26^ Furthermore, the pandemic prevented some of the pharmacists from being able to complete the HC-PCP within the 3-month period originally outlined in the protocol and extensions had to be provided. However, despite these limitations, the study results still demonstrated a statistically and clinically significant improvement in systolic BP, suggesting perhaps the results would be greater without these major limitations. These challenges were not unique to our study, as similar ones were reported by O’Reilly et al.,^27^ who evaluated the effectiveness of a community pharmacist-led support service for people living with severe or persistent mental illness. They identified that the pandemic created challenges for timely training and recruitment of pharmacies. As such, they switched their training to an online format and extended their recruitment period. Moreover, they allowed pharmacists to conduct follow-up visits by telehealth.^27^

In terms of generalizability, Alberta pharmacists have a full scope of practice, which includes independent prescribing and ordering laboratory testing. Thus, the HC-PCP may have varying results in other jurisdictions where pharmacists’ scope of practice is different. For practical reasons, patient follow-up was only 3 months, which may not fully capture the temporal-based nature of the impact of pharmacotherapy and other HC-PCP intervention benefits on BP. Future studies should investigate the impact of the HC-PCP over a longer follow-up period. Last, it is possible that there was selection bias, with pharmacists having a personal interest in the area of hypertension and thus increasing the likelihood of participating in the trial. This could have resulted in contamination of the control group, as pharmacists could have provided above-average hypertension care to their patients during the control period. Future studies could consider using the CSQ at multiple time points to allow a baseline comparison of satisfaction.

## Conclusion

Pharmacist care after taking the HC-PCP resulted in significantly improved BP, with a high level of patient satisfaction. This is unique, since most education programs are not evaluated at the patient level. The HC-PCP could be scalable to improve detection and control of hypertension in Canada. ■
